# Expedition into Exosome Biology: A Perspective of Progress from Discovery to Therapeutic Development

**DOI:** 10.3390/cancers13051157

**Published:** 2021-03-08

**Authors:** Arif Tasleem Jan, Safikur Rahman, Raied Badierah, Eun Ju Lee, Ehab H. Mattar, Elrashdy M. Redwan, Inho Choi

**Affiliations:** 1 School of Biosciences and Biotechnology, Baba Ghulam Shah Badshah University, Rajouri 185234, India; atasleem@bgsbu.ac.in; 2 Department of Botany, MS College, BR Ambedkar Bihar University, Muzaffarpur, Bihar 842001, India; shafique@ynu.ac.kr; 3 Biological Sciences Department, Faculty of Science, and Laboratory University Hospital, King Abdulaziz University, P.O. Box 80203, Jeddah 21589, Saudi Arabia; rbadierah@kau.edu.sa (R.B.); emattar@kau.edu.sa (E.H.M.); 4 Department of Medical Biotechnology and Research Institute of Cell Culture, Yeungnam University, Gyeongsan 38541, Korea; gorapadoc0315@ynu.ac.kr

**Keywords:** antigen, cancer, exosomes, immune response, therapeutics

## Abstract

**Simple Summary:**

Exosomes symbolize membrane-enclosed entities of endocytic origin. They play an important role in the intracellular communication by shuttling proteins, nucleic acids, etc., between cells of different tissues and organs. Recent studies have revealed an interplay between cell and exosomes; thereby highlighted their importance in disease diagnosis and possible implication for use in therapeutics. They are currently been explored for the strategic development of platforms towards their employment in achieving the target specific delivery of therapeutics. This review summarizes the composition, biogenesis and trafficking of exosomes in different cellular backgrounds and explores their multifarious role as drug delivery vehicles towards achieving correct functionality and efficacy of the therapeutic molecules. Additionally, it discusses genetic engineering platforms for employment in the designing of optimal delivery modules for their application in the delivery of drugs as part of anticancer therapy.

**Abstract:**

Exosomes are membrane-enclosed distinct cellular entities of endocytic origin that shuttle proteins and RNA molecules intercellularly for communication purposes. Their surface is embossed by a huge variety of proteins, some of which are used as diagnostic markers. Exosomes are being explored for potential drug delivery, although their therapeutic utilities are impeded by gaps in knowledge regarding their formation and function under physiological condition and by lack of methods capable of shedding light on intraluminal vesicle release at the target site. Nonetheless, exosomes offer a promising means of developing systems that enable the specific delivery of therapeutics in diseases like cancer. This review summarizes information on donor cell types, cargoes, cargo loading, routes of administration, and the engineering of exosomal surfaces for specific peptides that increase target specificity and as such, therapeutic delivery.

## 1. Introduction

Extracellular vesicles (EVs) represent a heterogenous population of membranous structures of varying sizes and cellular origin [1]. Their secretion into the extracellular milieu provides a means of mediating intercellular communication. Exosomes are a subset of EVs that were introduced to the scientific world as vesicles released from mature blood reticulocytes expressing transferrin receptor [2]. Exosomes develop intracellularly as multivesicular bodies (MVBs) that undergo fusion with cell membrane for their release into the extracellular space [3,4]. Exosomes are homogenous in shape with size ranging in between 30 to 150 nm compared to microvesicles and apoptotic bodies that exhibit substantial variation in size (from 100 to 1000 nm and 50 to 500 nm, respectively) [5,6]. They were initially determined to be definite intracellular entities by electron microscopy (EM) [7].

Morphologically, exosomes are “saucer-like” or “deflated football shaped” in whole-mount EM images, though their collapsed appearance is probably caused by sample preparation procedures, as SEM (scanning electron microscopy) showed them to be perfectly spherical [8]. Irrespective of their cellular background, exosomes display specific components on their surface and sequester molecules such as nucleic acids, cytokines, and other bioactive compounds. Following their secretion by epithelial, endothelial, and cells of other sources [9], exosomes make their way into body fluids such as blood, bile, bronchoalveolar lavage, urine, and breast milk [10,11,12], and their transport to distant sites facilitate cell-to-cell communication that influence physiologies and pathologies [13]. In addition to their role in intercellular communication [8], their potential diagnostic and therapeutic applications are of great interest to researchers. The present review was undertaken to provide an overview of the composition, biogenesis, and trafficking of exosomes, and to provide insight into the marked changes they undergo in diseased state and a detailed summary of their therapeutic applications with respect to types of cells and therapeutic cargoes, methods of loading, and possible administration routes. In addition, we discuss methods used to engineer exosomes with enhanced specificities and their current therapeutic statuses in the context of different diseases.

## 2. Composition

**Figure 1:**
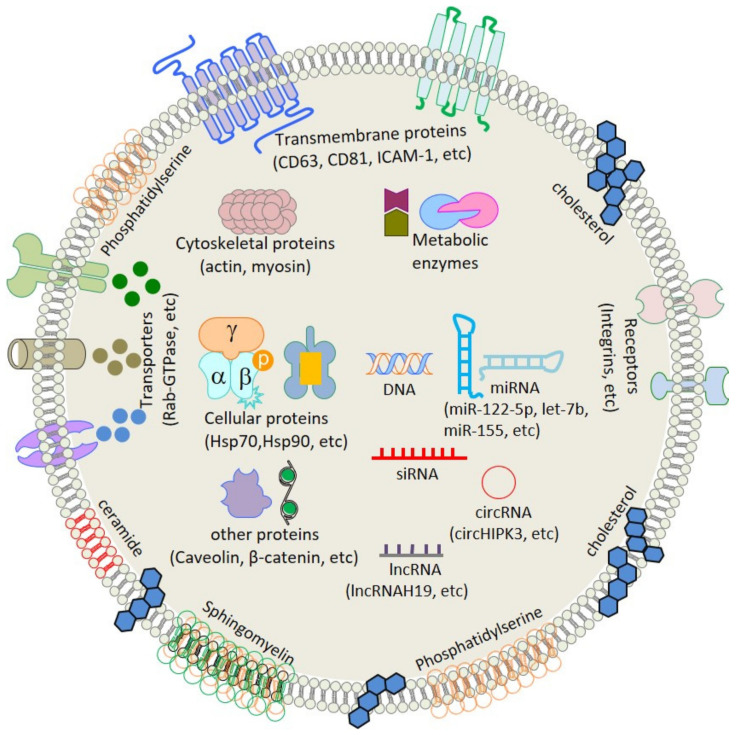
General representation of the exosome structure.

Exosomes constitute a subcomponent of the secretome [14], and their composition is dictated by the functional status of the cell (rested, stimulated, transformed, or stressed) [13]. Although the composition of exosomes are highly dependent on their origin, they all contain specific sets of endocytic proteins and nucleic acids (DNA, RNA), and are enclosed by a membrane of plasma membrane origin (Figure 1).

A wide range of methods are employed to separate exosomes from cell culture and body fluids (Table 1). Analyses of their composition by fluorescence-activated cell sorting (FACS), Western blot, and mass spectrometry have revealed them to have a series of tetraspanins (CD9, -26, -58 and others), RAB proteins, heat shock proteins (Hsp70, -90), endosome-associated proteins (Alix, TSG101), annexins, cytoskeletal elements (actin, tubulin), the lysosomal protein (Lamp2b), and the intercellular adhesion molecule (ICAM-1) and co-stimulatory molecules of T-cell origin such as CD86 [15,16,17,18]. Surface proteins such as heat shock protein, α4β1 (surface localized protein) on reticulocytes, A33 on enterocytes, and P-selectin on platelets are signatures of cell-specific exosomes [19,20,21]. Proteomic analyses of exosomes have shown them to possess surface-anchored sheddases, such as ADAM (a disintegrin and metalloproteinase), matrix metalloproteinases (MMPs), and MHC II molecules [22,23,24]. 

In addition to their role in extracellular matrix (ECM) remodeling, MMPs have been associated with intra- and intercellular communication via the proteasomal processing of exosome contents [25]. Enzymatic proteins, such as pyruvate kinases and peroxidases, have also been reported in human dendritic cells (DCs) and enterocyte-derived exosomes. In addition to displaying an array of intracellular proteins, exosomes contain DNA, and a wide range of non-coding RNAs (miRNAs, lncRNAs, and circRNAs). lncRNAs have emerged as regulatory RNA molecules with functions often related to cell differentiation and cell cycle regulation, whereas circRNAs act as competitive inhibitors of miRNAs during regulation of protein function [12,26,27,28]. Furthermore, exosome membranes are rich in lipids such as phosphatidylserine and cholesterol [29]. At the time of writing, the exosome database (http://www.exocarta.org; accessed on 20 December 2020) contained 9769 entries for proteins, 3408 for mRNAs, 2838 for miRNAs, and 1116 lipid entries. The presence of such a wide range of proteins, mRNAs, and miRNAs suggest enormous heterogeneity in terms of exosomal contents, the local expression of proteins and lipids, and the uniqueness of exosomes.

## 3. Biogenesis

The most accepted model of exosome biogenesis involves membrane orientation and inward budding. According to this model, budding events during exosome formation occur in a reverse membrane orientation, similar to that observed during apoptosis [22,46,47] and the release of milk fat globules from the epithelial cells of mammary glands [48]. Budding events during exosome formation involves phosphatidylserine flipping from the inner to the outer plasma membrane leaflet. Furthermore, electron microscopic observations have revealed the fusion profiles of late endosomes with the plasma membrane of antigen-presenting cells (APCs [15]), cytotoxic T-lymphocytes (CTLs [49]), dendritic cells (DCs [50]), and platelets [51]. Exosome production occurs in an active or passive manner, that is, with or without protein involvement. Active production involves a heterooligomeric protein complex referred to as endosomal sorting complex required for transport (ESCRT) and fusion of multivesicular bodies (MVBs) with the plasma membrane to enable exosome release. Ubiquitination is one of the sorting mechanisms that results in the incorporation of endosomal proteins into MVBs. The loading of monoubiquitinated entities into MVB compartments is achieved by four different ESCRTs (ESCRT- 0, I, II, and III) that interact with accessory proteins such as Vps-4 (vacuolar protein sorting-4) and ALIX (programmed cell death 6 interacting protein, also called PDCD6IP) [52,53,54]. A complex comprising ESCRT-0, HRS (hepatocyte growth factor regulated tyrosine kinase substrate), and STAM1 (signal transducing adapter molecule 1) aids in the recognition of ubiquitinated transmembrane proteins for incorporation into endosomal membrane [54]. Reportedly, ESCRT-I and II recruitment drive membrane budding, whereas ESCRT-III is required for bud scission [54,55,56]. The recruitment of ESCRT-III by ESCRT-I and II occurs with the involvement of ALIX, a protein that causes simultaneous binding of ESCRT-III to TSG101 (tumor susceptibility gene 101 and a component of ESCRT-I) [57]. After exosome membrane formation, ESCRT dissociates from MVB membrane and contributes to the transport of new cargos. ATPase VPS-4 (adenosine triphosphatase vacuolar protein sorting-4) is required for the dissociation of ESCRT from MVB membrane, which represents the first step of the ESCRT recycling machinery [54,58].

**Figure 2:**
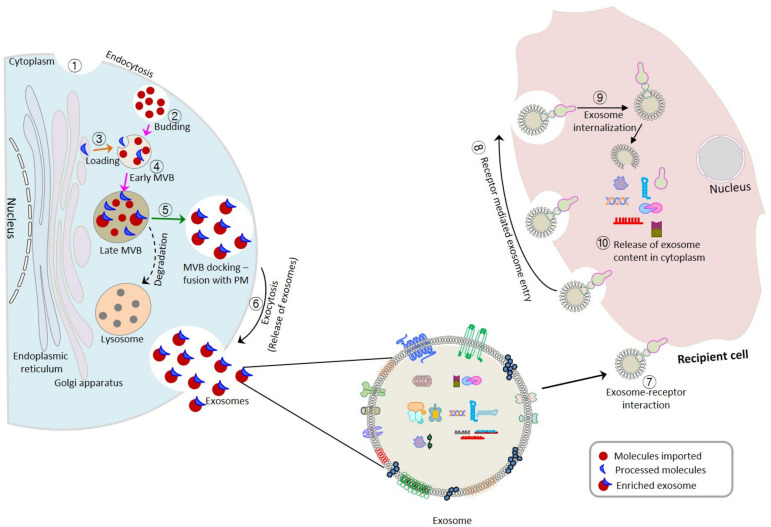
Exosome biogenesis and uptake at recipient surfaces.

The production of exosomes involves ten stages; (1) endosomal membrane invagination, (2) budding of intraluminal vesicles (ILVs), (3) loading of different entities (DNA, non-coding RNAs, proteins, etc.), (4) formation of multivesicular bodies (MVBs; ESCRT-0,I, II, & 4, Vps-4, ALIX), (5) docking and fusion of MVB that have escaped fusion with lysosomal components to the plasma membrane (PM; Rab and SNARE proteins), (6) release of exosomes into the extracellular milieu, (7) exosome-receptor interaction, (8) receptor-mediated exosome uptake by the recipient, (9) exosome internalization, and (10) release of exosome contents in cytoplasm (Figure 2).

Passive exosome formation involves the participation of lipids (ceramide), tetraspanins (CD63), and heat shock proteins independently of ESCRT [59,60,61]. Studies have shown localization of lipid metabolizing enzyme sphingomyelinase (SMase) and phospholipase D2 (PLD2) to MVB membrane induces the inward curvature required for exosome formation [62,63,64]. Concomitant inactivation of different ESCRT components using RNAi helped in establishing the independent nature of exosome biogenesis, as knockdown of different ESCRT components did not affect CD63 accumulation or suppress MVB formation [65,66]. Studies by Wehman et al. painted a mixed picture of this RNAi-based strategy as ESCRT- 0 and I silencing were found partially suppressing the shedding, but have no effect on ESCRT- II or III [67]. The dependence or independence of exosome biogenesis on the ESCRT machinery has been extensively studied and discussed elsewhere [56,68,69].

## 4. Exosome Trafficking

Fusion of MVBs with the plasma membrane results in the release of exosomes into the extracellular milieu. Although the mechanism that drives this fusion is unknown, the secretion of acetylcholinesterase tagged exosomes from reticulocytes was found to depend on the function of VAMP-7 (vesicle associated molecular pattern-7) [70]. Recent studies on exosomes carrying WNT3A morphogen revealed that their release is dependent on R-SNARE (soluble N-ethylmaleimide sensitive fusion attachment protein receptor) protein (also called Ykt6) [71,72,73]. Furthermore, MVB–plasma membrane fusion was found to be mediated by a ternary SNARE (t-SNARE) complex formed by v-SNARE (vesicle SNARE) and t-SNARE [73,74,75,76,77]. After the two membranes make contact, the energy barrier required for their fusion is overcome by the SNARE complex due to its association with the V_0_ subunit of V-type ATPase. The ability of V-type ATPase to overcome this energy barrier was found to be independent of its proton pump activity [78]. Other key regulatory components of the exosome secretion pathway include Rab proteins, e.g., Rab11 and Rab27b, which play key roles in the docking of MVBs to the plasma membrane [79].

Exosomes are rich in Rab GTPases, particularly Rab4 and Rab5, which are believed to be regulators of membrane trafficking [80]. Raposo et al. reported that plasma membrane fusion with MHC-II enriched MVBs in B-lymphocytes results in exosome release [15], and Zitvogel et al. reported stimulation of T-cell response by the components of exosomes from DCs [81]. Savina et al. deciphered the presence of Rab11 in exosome secretions [82], and in another study, though calcium transients were found to trigger exosome release, Rab 27 and Rab35 acted as regulatory GTPases for exosome secretion [83,84,85,86,87,88]. In addition, Alix and Vps4 (components of the ESCRT pathway) were reported to play an important role in exosome secretion [89], which was found to be regulated by P2X receptor activation by LPS-induced ATP on monocytes and neutrophils, and by TLR4 activation on dendritic cells [9,10,79,90].

## 5. Immunomodulatory Effect of Exosomes

**Figure 3:**
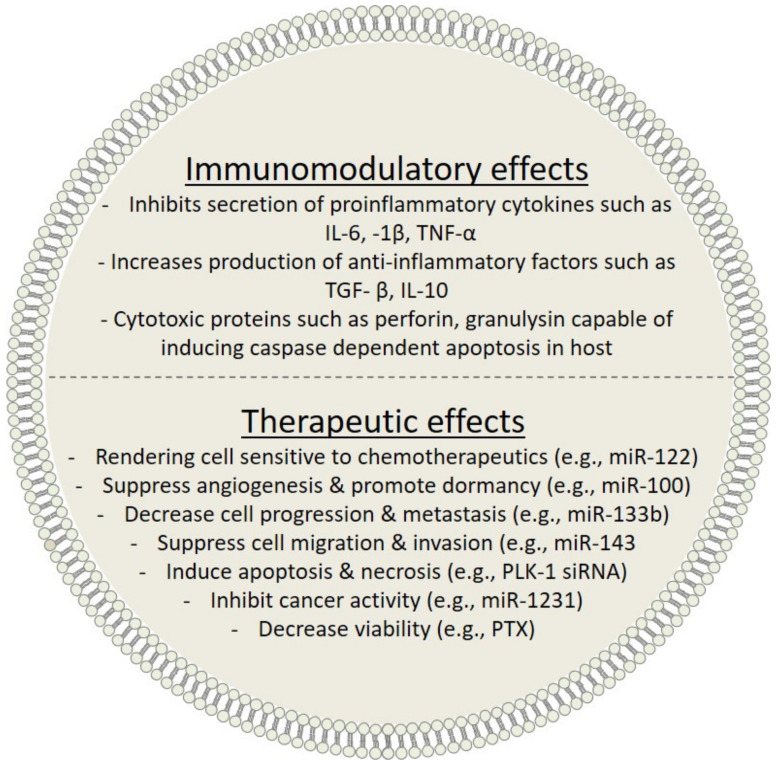
Immunomodulatory and tumor inhibitory effects of exosome loaded with different therapeutic cargoes.

Insights of the role of exosomes have revealed their importance as regulator of different biological processes under physiologic and pathologic conditions. Exosomes release into the extracellular milieu influences cellular morphology by interfering with cell signaling components and by modulating recipient gene expressions and functions and the cell differentiation program. Exosomes have been reported to influence infections [91,92,93], tumor development and metastasis [94,95,96,97,98], neurodegenerative diseases [99,100,101,102], inflammation, and autoimmune disorders [103,104,105,106]. In addition, they play crucial role in intracellular communication and in the pathogenesis of several diseases as they can transfer signals (cytokines, proteins, lipids, nucleic acids, and infectious agents) from cells to nearby or distant locations [91,107,108]. In one study, exosomes derived from immunocytes were found to contain a minimum of 98 immunogenic molecules [109]. The immunological functions of exosomes are highly dependent on their membrane proteins and cells of origin, and their stabilities in the extracellular space enable them to carry cargoes to distant cells [110]. Furthermore, the regulatory effects of exosomes involve cross-talk between different immune cells, for example, between B-lymphocyte-derived exosomes and CD8^+^ cytotoxic cells [111] and between T-cell-derived exosomes and DCs [112,113,114,115,116,117,118]. Here, we summarize the involvements of exosomes derived from mesenchymal stem cells (MSCs) and immune cells in cell-to-cell communication and immune system stimulation and suppression (Figure 3).

### 5.1. MSC-Derived Exosomes

Mesenchymal stem cells (MSCs) are multipotent stromal cells sourced from bone marrow, adipose tissues, placenta, or umbilical cord (Table 2). Their regenerative capacities underlie their importance in immune modulation [106,119,120,121,122]. The immunomodulatory effects of MSC-derived exosomes on peripheral blood mononuclear cells (PBMCs) have been well established. Exosomes from healthy human bone marrow are essential for the interaction between MSCs and PBMCs. Furthermore, MSC-derived exosomes can modulate the activities of lymphocytes, macrophages, neutrophils, DCs, and natural killer (NK) cells [123]. The ability of MSC-derived exosomes to inhibit the secretion of pro-inflammatory cytokines such as interleukin-6 (IL-6), interleukin-1beta (IL-1β) [124], and tumor necrosis factor-alpha (TNF-α) and to increase the production of anti-inflammatory factors such as transforming growth factor-beta (TGF-β) and interleukin-10 (IL-10) have been well described [125]. In addition, MSC-derived exosomes induce conversion of T-helper-1 (Th1) to T-helper-2 (Th2) cells and reduces potential of T-cells to differentiate into effector T-cells (Th17, capable of producing IL-17). The exosomes induce the proliferation and differentiation of CD4^+^ cells into Th2 cells, and thereby, suppress differentiation of Th1 to Th17 cells, which are known to participate in autoimmune response. Furthermore, an increase in the regulatory T-cells (Tregs) was also observed in the interaction between Th-cells and exosomes. Together, studies have revealed that MSC-derived exosomes have favorable immunomodulatory properties [106,120,121,122,123,124,125,126], and thus, they are considered as potential therapeutic candidates in many pathological contexts and as a convenient means of delivering therapeutics, enzymes, and genes to targeted cells [127]. Interestingly, recent evidence suggests that MSC-derived exosomes offer a potentially safe means of treating graft-versus-host disease (GvHD) [128].

### 5.2. DC-Derived Exosomes

Exosomes secreted by immune cells such as mature DCs displaying MHC molecules on their surface can act as antigen-presenting vesicles, thereby activate lymphocytes and initiate innate or adaptive immune responses [118,134,140]. DC-derived exosomes can bind antigenic peptides either by direct capture or by indirect antigen processing through parent DCs [141]. DC-derived exosomes displaying MHC II molecules mediate CD4^+^ helper cell activation by interacting with lymphocyte function-associated antigen 1 (LFA-1) expressed on the surface of T-cells [142]. In the context of antigen-presenting properties, DC-derived exosomes have greater immunostimulatory effect than intact DCs [143], and in the absence of antigen-presenting cells (APCs), exosomes can activate CD8^+^ lymphocytes, which supports a report that exosomes contain high levels of class I MHC proteins and ICAM-1 [110]. On the other hand, immature DC-derived exosomes have opposite effects on the immune system, as their cargoes are enriched with self-antigens and anti-inflammatory factors that might promote or induce immune tolerance. The immature DC-derived exosomes were also found to contain low levels of MHC II and co-stimulatory CD86^+^ molecules, and thus, were incapable of inducing immune response and instead had immunosuppressive effects [104,135]. In the background of allograft transplantation, immature DC-derived exosomes have been shown to promote allograft survival by secreting anti-inflammatory cytokine IL-10, and thus, suppressing T-cell proliferation [144]. It appears that DC-derived exosomes participate in the modulation of helper and cytotoxic T-cell immune responses, and thus, maintain immune tolerance.

### 5.3. NK-Derived Exosomes

NK cells are innate immune cells that play a central role in immune response. These cells exhibit natural cytotoxicity that enables them to lyse malignant and virus-infected cells without prior sensitization [145]. Also, activated NK cells can mediate immune response indirectly by secreting pro-inflammatory cytokines and chemokines that modulate adaptive cell-mediated immune response [146]. It has also been reported NK-derived exosomes have anti-tumor effects similar to those of NK cells [136]. In a recent study, activated NK cell-derived exosomes loaded with cytotoxic proteins, such as perforin (PFN), granulysin (GNLY), and granzymes (Gzm-A and Gzm-B) induced caspase-dependent apoptosis on entry into target cells [137]. A comparative study on the effect of resting and activated NK cells on tumor cells revealed that activated NK cell-derived exosomes contain high levels of FasL (Fas ligand) and perforin molecules with cytotoxic lysing activity against cancer cells, especially in hematologic malignancies, such as leukemia and lymphoma [147]. Furthermore, it has been suggested that understanding of the cytotoxic activities of NK-derived exosomes at the molecular level would undoubtedly aid in the development of immunotherapeutic strategies for the treatment of cancers and viral infections [148,149,150].

### 5.4. Treg-Derived Exosomes

Treg cells (suppressive T-cells) compose a subset of T-cells that play crucial immunomodulatory role by maintaining self-antigen tolerance and in preventing autoimmunity by inhibiting the proliferation of effector T-cells (i.e., CD4^+^ and CD8^+^ cells) [151]. Like other immune cells, Treg cells are capable of releasing exosomes, which markedly outnumber those released by other T-cell subpopulation [152,153,154]. The secretion of exosomes by Treg cells is highly dependent on hypoxia, calcium levels, and IL-2 [155,156,157]. Recent studies on the proteomic profile of Treg-derived exosomes have shown that these exosomes contain most components of the parent cell and transport several molecules such as miRNAs, CD73^+^, CD25^+^, and CD125^+^ (also known as cytotoxic T-lymphocyte-associated protein 4 (CTLA-4)) with marked immunomodulatory effect [139,158]. 

Recently, Treg-derived exosomes were reported to be enriched with miRNAs (e.g., miRNA-155, Let-7b, and Let-7d) as compared with parental Tregs, and when transferred to conventional effector cells, these specific miRNAs suppressed IFN-γ production and the expression of effector genes, thereby, inhibited T-cell proliferation [139]. An analysis of the Treg-derived exosomes showed high expression of CD73^+^, which perform an essential function in immune modulation by enhancing the production of adenosine (an anti-inflammatory modulator) that potently suppresses the proliferation and function of T-cells and block the production of IFN-γ and IL-2 [158].

## 6. Exploiting Exosomes for Therapeutics

The utilization of exosomes as drug delivery vehicles requires proper understanding of their production in different cellular backgrounds to achieve correct functionality and efficacy of the therapeutic cargoes. The following section summarizes the considerations that should be borne in mind to achieve targeted drug delivery.

### 6.1. Choice of Cells

In addition to stability in body fluids, reduced immune-stimulatory activity and minimal inflammatory response are prerequisites of therapeutic exosomes, and correct donor cell choice is a steppingstone toward achieving these developmental targets (Table 3).

Human cell lines such as HeLa and HEK293 and murine melanoma cell lines like B16-F1, B16-F10, and B16-BL6 are commonly used to produce exosomes [168,171,172,173,174,175,176,177,178,179]. In terms of immunogenic properties, immature DCs acts as a suitable donor cell alternative for exosome production [135]. Additionally, surface modification of locally expressed peptides enable exosomes to be used for targeted drug delivery [165,167]. DC-derived exosomes engineered to locally express rabies virus glycoproteins have been utilized to deliver siRNA across the blood-brain barrier in murine models [165]. However, despite their attractive characteristics, production at large-scale for clinical use is restricted due to technical difficulties [167]. To scale up production for clinical use, MSCs offer a possible alternative as they produce large number of exosomes [160,161,180,181,182]. The use of MSC-derived exosomes to deliver drugs to glioblastoma (GBM) xenograft tumors significantly reduced tumor size [161]. Although exosomes provide a platform for developing new therapeutic strategies, scale-up of MSC-derived exosome production is mostly hampered by technical difficulties [183,184], and manufacturing challenges remain to be properly addressed [7]. In this regard, a combination of tissue-specific targeting and scalability to large-scale production appears to be an appropriate developmental target.

### 6.2. Choice of Therapeutic Cargoes

Several therapeutic cargoes have been loaded into exosome-based delivery systems. Utilization of the abilities of exosomes to carry interfering RNAs [185,186] and deliver therapeutic cargoes offer a potential means of treating different cancers [187]. Several research groups have investigated the use of exosomes to carry siRNA for gene-based therapy [165,174,176,187,188,189,190]. Exosome-mediated delivery of siRNA not only reduces the risk of degradation, but substantially increases bioavailability and delivery efficiency. When MAPK1-siRNA was delivered using plasma or cell-based exosomes, a significant reduction in MAPK1 gene expression was observed in peripheral blood mononuclear cells [174]. In fibrosarcoma cells, gene knockdown by exosome-mediated delivery of RAD51 or RAD52-siRNA reduced viability and proliferation [176]. In a similar study, exosomes carrying the siRNAs of glyceraldehyde-3-phosphate dehydrogenase (GAPDH; the housekeeping gene) or β-site APP cleaving enzyme -1 (BACE1; an Alzheimer’s disease-associated gene) downregulated targeted protein level in neurons [165]. Also, the risk of hepatitis C virus (HCV) infection was reduced in liver cells treated with exosomes containing short hairpin RNAs (shRNAs) against viral entry receptor and the replicative machinery of HCV [49,176].

Dysregulation of the expression profiles of miRNAs is a characteristic of a large number of cancers [191,192], and subsequent studies reported that the exosome-based targeted delivery of miRNAs suppressed symptoms in different disease models [185]. Encapsulation of miR-150 in exosomes suppressed T-cell populations and reduced endothelial cell migration, and treatment of T-cells with the conditioned media of miR-122 transduced HEK293T cells increased miR-122 gene expression several-fold and suppressed hepatic inflammation, necrosis, and fibrosis [172,193,194]. Exosome-based delivery of miR-214 to hepatic stellate cells suppressed fibrosis by downregulating CCN2 expression [195,196], and miRNAs had tumor-suppressive effects when miR-143 or let-7a were transported to prostate and breast cancers in vivo [168,173]. However, no effect was observed when normal prostate epithelial cells were treated with exosome-encapsulated miR-143 [173]. MSC exosome (MSC^exos^)-mediated delivery of miR-133b was found to be effective for treating brain ischemia in mice [182], and exosome-mediated miRNA transfer from activated immune cells effectively induced epigenetic changes that influence convalescent plasma response to virus in COVID-19 [197].

In a systematic review, Khalaj et al. [198] reported that exosomes extracted from mesenchymal stem cells derived from bone marrow or umbilical cord ameliorate lung injury in experimental models by (1) attenuating inflammation (reducing pro-inflammatory cytokine levels, neutrophil infiltration, and macrophage polarization); (2) regenerating alveolar epithelium (by reducing apoptosis and stimulating surfactant production); (3) reducing microvascular permeability (by upregulating endothelial cell junction protein levels); and (4) preventing fibrosis (reducing fibrin production). The authors attributed these differential effects to the release of EV cargoes and identified several of the factors responsible, which included miRs126, -30b, -3p, -145, -27a-3p, syndecan-1, hepatocyte growth factor, and angiopoietin-1 [198]. Exosomal delivery of miR-146b inhibited tumor growth in a xenograft model of GBM [161,199], and the delivery of anti-miRs against miR-9 (an oncogenic miRNA) to GBM cells increased their susceptibility to chemotherapeutics like temozolomide [160]. The desired output highlights the communicative role played by exosomes in interaction between MSCs and GBM cells irrespective of the presence of gap junctions. These observations show that the exosomal delivery of miRNAs offers a promising means of delivering anti-cancer and anti-COVID-19 agents. Nevertheless, knowledge of the mechanisms of miRNA loading into exosomes would undoubtedly improve results. In particular, we suggest investigations be conducted to identify and characterize the EXO-motifs that direct the targeted exosome-based deliveries of miRNAs.

Exosomes containing chemotherapeutics like doxorubicin have shown growth inhibitory effect on xenografted breast and colon adenocarcinoma tumors [167,200]. Enhancement in the efficiency of chemotherapeutic agents like doxorubicin achieved by direct delivery of immature DC-derived exosomes effectively reduced side effects on non-targeted organs, especially the heart [167,201]. An exosome preparation of JSI-124 (a STAT3 inhibitor) effectively reduced tumor volume in a murine model of GBM [170,202,203], and notably, exosomes containing 5-fluorocytidine (a prodrug) facilitated its conversion to 5-fluorouracil and 5-fluoro-deoxyuridine and resulted in tumor cell apoptosis in an orthotopic model of schwannoma [175,204]. Furthermore, an exosome-based co-treatment offer another means of treating malignancies, and exosomes loaded with super paramagnetic iron oxide nanoparticles (SPIONs) were shown to have potential use as an MRI cancer imaging agent [177,205].

### 6.3. Exosome Loading Procedures

The loading of therapeutic cargoes into exosomes involves the use of classical incubation and electroporation methods and transfection reagents and the modern techniques of donor cell transfection or activation [177,188]. However, simple incubation with a cargo is sometimes sufficient to load exosomes (Table 3). The best example of this is provided by curcumin, a natural compound with an anti-inflammatory effect, which can be loaded by simple incubation for 5 min at 22 ℃, presumably because curcumin rearranges membrane lipids and alters membrane fluidity [206,207]. The encapsulation efficiency for the drug doxorubicin was higher for exosome-mimetic bioengineered nanovesicles generated from filtered monocytes or macrophages [194,200]. On the other hand, the loading of small-sized cargoes, such as miR-150, was efficiently achieved by simple incubation [200,208].

Efficient loading of therapeutic cargoes into exosomes can also be achieved by electroporation at 150–700 V [165,186], but the effectiveness of cargo loading depends on the donor cell type [167,174,176], exosome type, and cell concentrations [165,167,177,209]. Quantification of cell delivery using fluorescently labeled siRNA revealed higher uptake than by chemical reagent-based transfection [163,174]. An analysis of the cell viability after electroporation of exosomes with therapeutic cargoes was used to investigate the efficiency of the technique [176]. Although it seems to be a suitable clinical option, electroporation is known to have adverse effects on the integrities of exosomes and cargoes, for example, it has been reported to induce exosome and siRNA aggregation. In fact, after optimizing delivery parameters and using trehalose medium to minimize exosome aggregation [177], siRNA retention in exosomes was only <0.05% [210]. Nevertheless, the loading of drugs like doxorubicin by electroporation is still considered a better option than incubation or chemically based transfection methods, because it better maintains the functionality of the drug [167]. The use of chemical-based transfection methods to load therapeutic cargoes such as siRNA into exosomes has restricted usage because they are less efficient than that achieved using HiPerFect transfection reagent-based methods [174,176]. Although Lipofectamine 2000-based siRNA loading was reported to alter gene expression in recipients, leftover micelles generated during exosome preparation prevented quantification of the effects of siRNA cargoes at target sites [174,176].

Transfection of donor cells with appropriate cargoes to obtain cargo-loaded exosomes appears to offer an acceptable means of therapeutic exosome production [211]. Destined for secretion, transfection of donor cells with the overexpression construct facilitates entry of therapeutic cargo into the lumen or its labeling to the surface of exosomes [161,168]. In most studies, miRNAs are transfected as overexpression constructs in miRNA expression vectors and then loaded into exosomes [161,162,172,173,195]. Exosomes produced from MSCs transfected with a construct carrying miR-146b were found to restrict tumor growth effectively [161]. In a similar study, let-7a containing exosomes with a surface expressed target peptide efficiently delivered cargo to epidermal growth factor receptor (EGFR) expressing breast cancer cells [168]. Elevated miR-214 expression achieved by transfecting cells with anti-miRs seems to be a promising alternative to transfecting donor cells with pre-miR-214 [160,195]. Though transfection of donor cells seems appropriate for exosome loading for in vivo studies, engineering cells to express desired surface molecules and carry maximum therapeutic load is time-consuming. Thus, non-autologous exosome producing methods are required to generate non-immunogenic exosomes with specific targeting characteristics for clinical use.

Studies that used activated donor cells to generate exosomes have shown them to be less appropriate choice for exosome production as they are capable of transferring therapy resistance to drug sensitive cells via, proteins, that increases DNA repair and tumor cell survival along with disposal of the pro-apoptotic proteins. Using this methodology, stimulation of THP-1 cells using inflammatory stimulants caused an increase in miR-150 levels in vesicles [193], and in another study, co-culture of brain extracts from rats that had undergone middle cerebral artery occlusion show increased miR-133b levels [182]. Hypoxia is a characteristic of tumor microenvironment [212,213,214] and is believed to enhance release of exosomes. Studies that used hypoxic condition to generate exosomes have revealed them to be enriched with CD81, CD63 and HSP70 markers [215,216,217]. Although hypoxic microenvironment alter the miRNA cargoes of exosomes from different cells [215], exosomes generated under hypoxic conditions were found to be enriched in IL-8 and IGFBP3 mRNAs and proteins, which promote the proliferation and migration of angiogenic cells in vitro [218,219].

### 6.4. Exosome Administration Routes

Conventional routes are required to administer drug-loaded exosomes. In addition, to the efforts being made to increase stability during long-term storage, research is also being conducted to identify means of delivering drugs to tumors located in fragile tissues [220,221]. Administration of exosome-based therapeutics via intravenous injection has been commonly used to deliver drugs to brain, pancreas, and tumors in other tissues [165,167,168,172,198,222,223,224,225,226], and the endogenic origins of exosomes help them escape removal by immune cells [227]. Exosome-based delivery of therapeutics increases drug stability and enables high drug loadings in body fluids [227], and lack of lymphatic drainage and the presence of fewer blood vessels aid in the retention of exosomes in tumorigenic tissues [12,228,229], which enhances their therapeutic efficacies. Upon administration through an intravenous mode, the half-life of exosome-based therapeutic cargo in circulation was approximately two minutes [178]. The distribution of exosomes to lungs, liver, spleen, and bone marrow and their later accumulation in liver and then lungs, suggests a clearance mode similar to that of synthetic liposomes [178,230,231]. Accumulation in liver has also been reported in studies on the administration of EGFR-bearing exosomes with high affinity for hepatic tissues, and in tumor tissues in a xenograft model of breast cancer [168,232]. Despite their exhaustion in circulation within short span of time, the presence of therapeutic cargo in tumor vasculogenesis appears to program bone marrow-derived MSCs [233]. In addition, modifications, such as PEGylation, aimed at increasing their half-lives, are still warranted [234].

The intra-tumoral injection (another appropriate administration technique) of exosome-encapsulated therapeutics for the treatment of different cancers resulted in successful reduction in tumor volumes [161,173,175,235,236]. The combined use of intratumoral injection and tumor resection further reduces the risk of tumor recurrence [161,237]. The oral administration of exosomes potently induce intestinal stem cell proliferation after stable passage through the gut in a murine model of colitis [238]. Administration of exosomes loaded intraperitoneally with curcumin increased their bioavailability by improving their stability in the circulation [170]. Intranasal administration of exosomes encapsulating curcumin or Stat3 inhibitor for delivery to microglial cells reduced inflammation in brain [202], and the subcutaneous administration of MHC II over-expressing exosomes proved effective in murine melanoma [179,202,239]. The exosomes loaded with therapeutic cargo exerts their effects at the target with in a short span of time after its delivery to the target [165,178]. Adoption of exosomes in clinical settings requires characterization of exosome protein compositions in order to avoid adverse effects in patients.

## 7. Increased Specificity by Exosome Engineering

The expression of targeting peptides or proteins on exosome surface is a prerequisite for the specific delivery of therapeutic cargoes and in avoiding the adverse effects associated with chemotherapeutic agents on normal cells surrounding tumors. Although many studies have been performed on the exosome-based delivery of therapeutic cargoes, few have addressed the engineering of exosomes to achieve the target-specific delivery of therapeutic cargoes [240,241,242,243,244]. The exosome-engineering aimed at inserting a peptide correctly into exosomes, while avoiding cleavage of peptide regions, is accomplished by expressing the target peptide as a fusion product with the surface localized lysosomal associated membrane protein-2b (Lamp-2b) [245,246]. This bioengineering approach helps to enhance the uptake of exosomes and as such treatment specificities in tissues of interest. An excellent example of this phenomenon is provided by RVG and iRGD peptides, which when engineered on immature DC-derived exosomes helps to target therapeutics to the brain and tumor tissues [165,167]. The expressions of hemagglutinin, myc-tag, and peptide (epidermal growth factor; EGF or GE11) as a fusion protein with platelet-derived growth factor receptor (PDGFR) on the surface of exosomes effectively targeted drugs to tumors [168]. With ability to bind specifically to EGFR-upregulated cells in tumor tissues, GE11-mediated delivery of therapeutic cargoes proceeds without activating the EGF-receptor [168], and thus, this method of delivery appears to be appropriate for treating different types of cancers [247].

U937 or Raw264.7 cell-derived exosomes or exosome mimetic nanoparticles expressing surface LFA-1 induced significant reduction in the tumor volume when used to deliver chemotherapeutics to tumor cells [200]. LFA-1 facilitates binding of exosomes to endothelial cell adhesion molecules and has been used to deliver therapeutics to rapidly growing tumors with extensive neovascularization [200]. The cell-specific characteristics of exosomes facilitate the delivery of therapeutics more specifically to tumor tissues. Transfection of the CIITA gene to induce the expression of MHC II in murine melanoma cells resulted in the production of exosomes expressing high surface levels of the MHC II protein [179]. The study indicated that MHC II has two functions, that is, as a targeting peptide to deliver cargoes to specific destinations and as a therapeutic [179]. Exosomes derived from choroid plexus epithelial cells expressing folate receptor-α (FRα) were reported to transport cargo to brain parenchyma cells after passaging through the choroid plexus [239]. The ability to cross the blood–brain barrier (BBB) or choroid plexus and the surface expression of targeting peptides on exosomes hold great promise for drug delivery to the brain [165,239,248]. The surface expression of tetraspanin proteins can be used as an alternative method to engineer exosomes that deliver therapeutics to tumor tissues [222]. Similarly, utilizing target specific antibodies to coat the surface of exosomes provides another means of avoiding the laborious procedure of modifying membrane proteins.

## 8. Advancement in the Therapeutic Uses of Exosomes

Many commercial enterprises have been established to exploit the exosome-based delivery of therapeutics. Codial BioSciences (Cambridge, MA, USA) has devised a specific platform called engEx™ for engineering exosomes to deliver different therapeutics entities [249]. exoSTING—a therapeutic entity developed on exosome backbone with minimal cytotoxicity is viewed as a promising therapeutic delivery candidate in the treatment of cancer [249]. Exosomes carrying therapeutic cargoes have also been subjected to clinical trials (Table 4). In a phase I study, DC-derived exosomes (DEX) loaded with MAGE3 antigenic peptides were administered to stage III/IV melanoma patients [250]. Studies performed on the intradermal and subcutaneous administration of DEX revealed an increased number of natural killer cells (NKCs) and reconstitution of NKG2D expression on NK and CD8^+^ T-cells. Autologous exosome production from these non-toxic cells was achieved successfully using standard manufacturing protocols [250]. In a phase II study of DC-derived exosomes (DEX2) loaded with the chemotherapeutic metronomic cyclophosphamide, DEX2 encapsulation increased the immunostimulatory effect of the drug on T-cells (NCT01159228). In addition, the application of ascites-derived exosomes (AEX) together with GM-CSF was found to have greater cytotoxic T-cell response in colorectal cancer than AEX alone [251]. Furthermore, exosome-based treatment was subjected to clinical trials in malignant glioma. Implantation of glioma cells isolated from resected tumor tissue into the abdomen of glioma patients treated with drug-inhibiting insulin-like growth factor receptor-1 (IGF-1) induced apoptosis in implanted cells, and this was followed by exosome release from these cells that stimulated the immune system to induce a T-cell mediating antitumor response (NCT01550523).

A joint venture between PureTech Health and Roche aimed at developing novel exosome technologies, led to the development of milk exosome-based technology for the oral administration of antisense oligonucleotides [252], and this technology is considered to have the potential to enhance treatment efficacies and reduce toxicities as compared with conventional intravenous injection. In addition, plant-derived exosomes were assessed for potential use as cancer treatments at the James Graham Brown Cancer Center. Orally administered exosomes containing curcumin were tested for therapeutic effectiveness against colorectal cancer (NCT01294072) and evaluated for their effects on oral mucositis and pain after chemotherapy for head and neck cancers (NCT01668849). These trials, which are ongoing and completed, respectively, have demonstrated good safety profiles in clinical settings, and relevance of continuing the development of exosome-based drug delivery systems.

## 9. Conclusions

Exosomes are considered as versatile carriers due to their immunogenic nature and abilities to traverse biological barriers (e.g., the blood–brain barrier) and migrate to tissues or areas with no blood supply (e.g., dense cartilage matrix). Exosomes encapsulate many cargo types (DNAs, RNAs, proteins, and lipids) and transport them via body fluids to nearby or distant cells. Their biocompatibilities and the genetic engineering possibilities that prevent unwanted exosome accumulation and enable selective targeting, have encouraged researchers to develop exosome-based drug delivery systems. Selection of the source and optimization of the isolation methods are currently being explored towards achieving enhancement in the production of exosomes with distinct characteristics and functionalities. Studies are currently being undertaken on the potential therapeutic use of exosome derived from human tissues as drug carriers. However, such investigations are hampered by lack of suitable isolation methods and drug uptake discrepancies. Currently, the use of hollow fiber-based bioreactors offer an attractive means of harvesting exosomes with reproducible characteristics. As effectiveness of therapeutic cargo depends on the source of generation of exosomes and its release at target site, efforts are required to understand exosome generation in different cellular backgrounds and their drug uptake at the target tissues. Exosomes exhibit a lipid bilayer structure with embedded characteristic surface protein signatures that promote uptake at target sites. Given the complexity of exosomes, internalization of exosomes loaded with therapeutic cargoes can be achieved by incorporating cell-penetrating peptides (CPPs), such as arginine-rich CPPs, which stimulate micropinocytosis at target sites, onto their surfaces. Investigations are required to determine the optimal dosage, administration methods, and kinetic characteristics, and to further investigate the effects of environmental conditions, such as pH, on the efficiency of cargo delivery. Moreover, comprehensive investigations of the properties of cells used for exosome production and the functionalities of exosomes are needed to ensure target-specific delivery of therapeutics in the context of personalized medicine. Furthermore, the standardization of large-scale production and purification procedures would undoubtedly improve exosome reproducibility and aid in the development of exosome-based cancer therapeutics. Finally, investigations aimed at elucidating the mechanisms that govern the specific delivery of exogenously administered exosomes, their biodistribution, and pharmacokinetics would help to achieve the developmental transition of exosomes to the clinical level.

## Figures and Tables

**Table 1:** Exosome isolation methods and their advantages and disadvantages.

Extraction Method	Advantages	Disadvantages	Reference(s)
Ultracentrifugation (UC; Differential centrifugation)	High Purity	Low yield, time-consuming, requires costly instruments	[30,31,32,33]
Density gradient centrifugation	Satisfactory purity	Low yield, time-consuming	[30,34,35]
Size elusion chromatography (SEC)	Relatively gentle	Unable to differentiate exosomes from particles of similar size	[35,36,37]
Filtration (Non-porous membrane-based)	Simple, time saving	Low yield, high contamination	[38]
Polymeric precipitation	High yield	Low purity than SEC	[39]
Affinity capture (Vn-96 peptide-based)	Simple and time-saving, high yield, high purity	Costly, unsatisfactory recovery	[40,41,42]
Immunoaffinity capture (Antibody-based)	Simple and time-saving, high yield, high purity	Costly, non-specificity of Abs	[43,44,45]

**Table 2:** Characteristics of exosomes derived from human mesenchymal stem cells (MSCs) and other human immune cells.

Source of Exosomes	Markers	Characteristic miRNAs	Cargo/Pathway	Role	Reference(s)
MSC-derived exosomes (BM-MSCs-exo, AD-MSCs-exo, UC-MSCs-exo, and PL-MSCs-exo)	CD9,CD34,CD44,CD63,CD81,CD90, CD105, ALix, TSG101, OCN, OPN, BMP-7, NKG2D ULBPs	miR-155, miR-146	P13K/AKT/AKT mTOR, TGF-β/Smad/β-catenin, STAT3/Bcl-2/Beclin1, IL-6, ERK1/2, P38, MAPK	Immunosuppressive	[129,130,131,132,133]
Mature DEXs Immature DEXs	CD63, CD81, CD82, αMβ2, MFG-E8	miR-155	Syntenin Gi2α, β-catenin	Immunostimulatory	[134]
	Annexins, CD63, Alix, TSG101, Calnexin and CCR-7	miR-125b-5p, miR-146a, and miR-148	Syntenin Gi2α, β-catenin	Immunosuppressive	[135]
NKC-derived exosomes (NK-exo)	CD56	miR-186, miR-328, miR-21, miR-29a	Granulysin (GNLY), TGF-β, granzymes (Gzm-A & Gzm-B), perforin (PFN)	Immunostimulatory	[136,137]
Treg-derived exosomes (Treg-exo)	CD25 and CTLA-4	miRNA-155, Let-7b, Let-7d	IL-10, IL-35, and TGF-β	Immunostimulatory	[138,139]

BM, Bone Marrow; AD, Adipose Tissue; UC, Umbilical cord; PL, Placenta; DEX, Dendritic cell derived exosomes.

**Table 3:** Exosomes in therapeutics. The table summarizes sources, cargoes, loading mechanisms, and effects observed for exosomes from different cell types.

Exosome Source	Cargo and Loading Mechanism	Effect Observed	Reference(s)
Mesechymal Stem Cell	miR-124 (Transfection)	Reduction of cell migration & self-renewal	[159]
	Anti-miR-9 (Transfection)	Reversal of chemoresistance	[160]
	miR-146b (Transfection)	Reduction of progression & metastasis	[161]
	miR-133b (Transfection)	Suppression of progression	[162]
	PLK-1 siRNA (Electroporation)	Induction of apoptosis & necrosis	[163]
	Paclitaxel (Incubation)	Growth inhibition of human pancreatic adenocarcinoma cell	[164]
Dendritic Cell	BACE1 siRNA (Electroporation)	Knockdown of specific gene after specific siRNA delivery to the brain for AD	[165]
	VEGF siRNA (Electroporation)	Suppression of tumor growth in breast cancer	[166]
	GAPDH siRNA (Electroporation)	Knockdown of specific gene after specific siRNA delivery to the brain for AD	[165]
	Doxorubicin (Electroporation)	Specific drug delivery to the tumor site & inhibited tumor growth	[167]
HEK293	Let-7a mimic (Transfection)	Target EGPR-expressing cancerous tissues with nucleic acid drugs for breast cancer	[168]
HEK293T	BCR-ABL siRNA (Transfection)	Overcome pharmacological resistance in CML cells	[169]
Mouse lymphoma cell	Curcumin (Mixing)	Increase anti-inflammatory activity	[170]

**Table 4:** Clinical trial data of exosomes used to treat various diseases.

Exosome Source	Condition	Payload	Phase, Patients	Clinical Trial Identifier
MSCs	Multiple organ failure	NA	NA (*n* = 60)	NCT04356300
	Severe COVID-19 Pneumonia	NA	Phase 1 (*n* = 24)	NCT04276987
	Periodontitis	NA	Phase 1 (*n* = 10)	NCT04270006
	Dry Eye	NA	Phase 1 (*n* = 27)	NCT04213248
	Type I Diabetes Mellitus	NA	Phase 1 (*n* = 20)	NCT02138331
	Metastatic Pancreatic cancer	KRAS G12D siRNA	Phase 1 (*n* = 28)	NCT03608631
	Macular Holes	NA	Phase 1 (*n* = 44)	NCT03437759
	Cerebrovascular disorders	NA	Phase 1/2 (*n* = 5)	NCT03384433
	Diabetic Nephropathy	Placebo	NA (*n* = 38)	NCT04562025
Dendritic Cell	Sepsis	Antibiotics	NA (*n* = 50)	NCT02957279
	Non-small cell lung cancer	Antigens	Phase 2 (*n* = 41)	NCT01159288
		MAGE tumor antigens		
	Metastatic melanoma	MAGE 3 peptides		
Plant	Colorectal cancer	Curcumin	Phase 1 (*n* = 7)	NCT01294072
	Obesity	NA	NA (*n* = 160)	NCT02706262
	Head & Neck cancer	Grape extract	Phase I (*n* = 60)	NCT01668849
	Polycystic ovary syndrome	Ginger & Aloe	NA (*n* = 176)	NCT03493984

Source: https://www.clinicaltrials.gov (accessed on 24 December 2020). NA = Not available.
